# A community feasibility study of a cooking behavior intervention in African-American adults at risk for cardiovascular disease: DC COOKS (DC Community Organizing for Optimal culinary Knowledge Study) with Heart

**DOI:** 10.1186/s40814-020-00697-9

**Published:** 2020-10-19

**Authors:** Nicole Farmer, Tiffany M. Powell-Wiley, Kimberly R. Middleton, Brenda Roberson, Sharon Flynn, Alyssa T. Brooks, Narjis Kazmi, Valerie Mitchell, Billy Collins, Rachel Hingst, Lucy Swan, Shanna Yang, Seema Kakar, Timothy Harlan, Gwenyth R. Wallen

**Affiliations:** 1 National Institutes of Health, Clinical Center, Bethesda, MD USA; 2 Social Determinants of Obesity and Cardiovascular Risk Laboratory, Cardiovascular Branch, Division of Intramural Research, National Heart, Lung, and Blood Institute, National Institutes of Health, Bethesda, MD USA; 3 Intramural Research Program, National Institute on Minority Health and Health Disparities, Bethesda, MD USA; 4 George Washington University School of Health Sciences, Washington, DC, USA

**Keywords:** Dietary behavior, Culinary medicine, Cooking intervention, CBPR, Feasibility study

## Abstract

**Background:**

Cooking interventions have increased in popularity in recent years. Evaluation by meta-analyses and systematic reviews show consistent changes in dietary quality reports and cooking confidence, but not of cardiovascular (CVD) biomarkers. Interventions evaluating or reporting behavioral mechanisms as an explanatory factor for these outcomes has been sparse. Moreover, evaluations of cooking interventions among communities with health disparities or food access limitations have received little attention in the literature.

**Methods:**

This study will occur over two phases. Phase 1 will assess acceptability among the target population of African-American adults living within an urban food desert. Phase 2 will consist of a 6-week cooking intervention delivered at a community kitchen setting. Pre and post intervention visits for clinical examinations and biomarker collection will be conducted, as well as dietary and cooking skill assessments. Primary outcomes include cooking behavior and feasibility measures. Secondary outcomes are related to dietary quality, psychosocial factors, CVD biomarkers, and food environment measures.

**Discussion:**

This study seeks to demonstrate feasibility of a community-based cooking intervention and to provide necessary information to plan future interventions that identify cooking behavior as an outcome of participation in cooking interventions among African-American adults, especially in relation to dietary and biomarker outcomes.

**Trial registration:**

This study was registered at ClinicalTrials.gov (NCT04305431) on March 12, 2020.

## Background

Disparities in cardiovascular disease (CVD) morbidity and mortality among African-Americans persist despite advances in risk factor identification and evidence-based management strategies [1]. Studies suggest risk factor reduction positively attenuates the mortality disparity between African-Americans and other racial/ethnic groups [1]. Closing the CVD disparity gap may require attention to more proximal causes of CVD that lower risk factors, including cardiovascular health behaviors such as dietary intake and physical activity.

Dietary behaviors consist of eating habits, food/nutrition choices, and meal preparation or cooking behavior. Among dietary behaviors, national survey data show those who frequently prepare meals at home have a better diet quality overall determined by higher vegetable intake [2] and lower daily calorie intake [3]. Observational studies show possessing a greater number of cooking skills is linked to healthier food choices [4]. However, cooking and meal preparation at home have decreased in the United States (U.S.) [5], while there has been an increase in consumption of food away from home [6]. Consequently, it is possible that the inability for Americans to meet the recommended guidelines for fruit and vegetable consumption [7] may be a public health consequence of the reduction in cooking at-home and shift to convenience foods.

The trend toward less cooking is also occurring in racial minority populations in the U.S., with African-Americans reporting cooking dinner at home less than other racial/ethnic populations [8]. An updated analysis of U.S. Adult Time Use survey data showed African-American men were the racial and gender group least likely to report engaging in meal preparation activities [9]. Our own group’s NHANES (National Health and Nutrition Examination Survey) analysis of cooking frequency among African-Americans found that employment, income, and self-perceived diet quality were significant determinants of home cooking frequency [2]. Cross-sectional studies from an African-American population in a Baltimore food desert show that self-efficacy and beliefs are also contributors and are positively associated with cooking frequency and type of home cooking method [10, 11].

When evaluating published cooking interventions, studies among African-Americans from faith-based and community settings show successful self-report of dietary quality for improved fruit and vegetable consumption and Dietary Approaches to Stop Hypertension (DASH) score post cooking interventions [12–15]. This is in contrast to nutrition education studies that allowed participants to make their own food for the DASH diet [16]. In these studies, African-Americans were less likely as compared to Non-Hispanic Whites to be adherent to the DASH eating plan [16]. The differing results between cooking intervention studies and free-living studies suggest an important role of cooking interventions on implementation of a cardiovascular protective dietary plan among African-Americans.

**Fig. 1:**
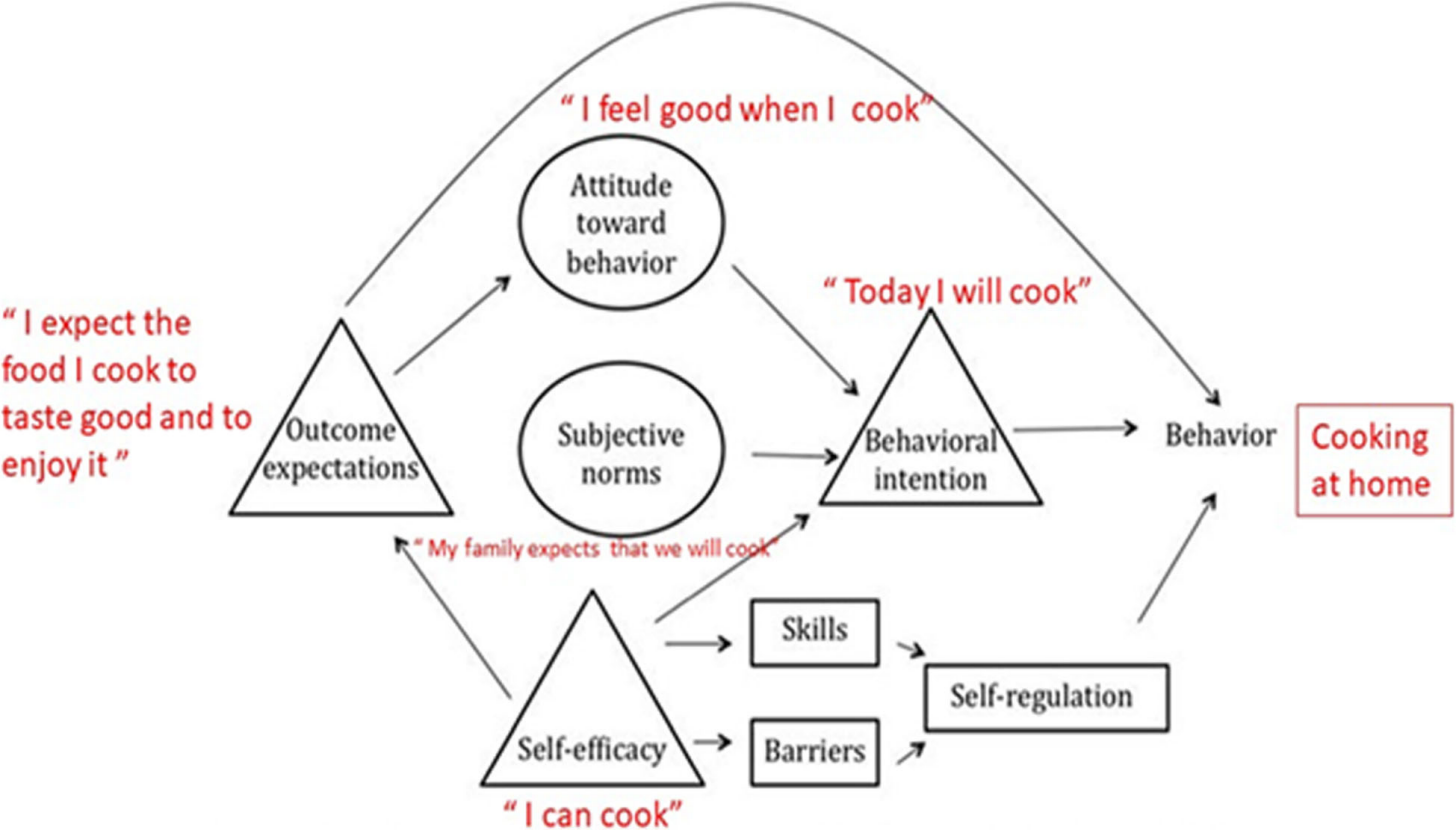
Diagram of cognitive processes and constructs involved in theory of planned behavior and social cognitive theory adapted for hypothesized cognitive processes involved in home cooking. Constructs from SCT are represented by rectangles. Constructs from TPB are represented by circles. Constructs common to both are represented by triangles. Adapted from Koenings M and Arscott S, 2013

Cooking can be defined as a goal-directed behavior, where the goal is to create a meal/dish to satisfy nutrition needs or tastes for oneself or others. Two theoretical frameworks related to goal-directed behaviors that pertain to current understanding of facilitators and barriers for cooking are the social cognitive theory (SCT) and the theory of planned behavior (TPB). Both theories include individual factors related to home cooking, including self-efficacy (Fig. 1). Self-efficacy is defined as a person’s belief in their capabilities to produce designated levels of performance that exert influence over events that affect their lives. It is an important construct in determining someone’s motivation for a health behavior change. In terms of cooking behavior, this can transfer into self-efficacy surrounding cooking tasks, such as preparing a meal or cutting up an ingredient, to shopping for particular food items, to determining meals based on desire to change or improve one’s health. These self-efficacy influences are built into cooking interventions that include aspects of personal agency, also known as food agency [17].

### Gaps in the literature

Recent systematic reviews of community level cooking interventions found significant improvements in attitudes, cooking self-efficacy, and healthy dietary intake and participation in cooking interventions [18, 19]. However, no significant outcomes are reported for cardiometabolic risk factors, including blood pressure and body mass index (BMI) within these reviews. Although methodological variation and high risk of bias from studies were identified, the findings are similar to earlier systematic reviews of cooking interventions [20]. The current literature thus leaves several questions to be addressed. If dietary intake changes and cooking efficacy changes are occurring, what is the behavioral pathway for these changes? Furthermore, what behavioral pathways may be needed or are sufficient for the expected biomarker changes to occur?

A combination of better informed food purchasing for home consumption, better choices when eating out, or more frequent home cooking may lead to dietary benefits, but ultimately may not explain needed or sufficient behavioral mechanisms. For example, practice and modeling are tenants of the SCT construct of self-efficacy. Participants may receive practice and modeling within intervention sessions that contribute to self-efficacy, but may not have these same opportunities transfer to the home environment if home cooking behavior is not the behavior mechanism. Thus, it is conceivable that if opportunities for practice only occur within the intervention, then long-term behaviors needed for cardiometabolic change may not occur. Despite this, most cooking intervention studies only include dietary quality or health outcome variables as the primary outcomes reported [21], not cooking behavior. Furthermore, identification of cooking behavior as a mechanism is important, as cooking has the potential advantage of being more economical [22] or conferring a psychosocial benefit [23] that may be of special benefit to communities disproportionately affected by diet related chronic diseases.

The current gaps in the literature around the role of behavior may also occur because assessing behavioral mechanisms for cooking intervention outcomes can be complex. For instance, the concept of cooking skills is representative of a confluence of skills: perceptual and conceptual, mechanical, academic (knowledge of preparing raw and cooked foods), and cognitive planning skills. Additionally, for each individual, the interplay between these skills may differ based on their intrapersonal or interpersonal experiences. Lastly, the absence of a detectable effect may not result from the cooking intervention being ineffective, but rather could be a consequence of the logistical difficulties in standardizing and evaluating complex health interventions, especially in an experimental process [24]. Ideal participation and engagement level in an intervention to achieve an increase in cooking frequency is not well defined in the literature. Furthermore, it is unclear if participation and engagement level is expected to vary between individuals, or by objectively determined baseline cooking skill.

Feasibility allows for focus on real-world acceptability and implementation, while involving key stakeholders (study participants) at each stage of the exploration process [25]. A feasibility study approach also allows for process evaluations that provide a detailed understanding of complex interventions by examining their implementation, mechanism of impact, and context [26]. In relation to facilitators and barriers to cooking, understanding aspects of feasibility could help inform behavioral mechanisms that can explain cooking intervention results. The purpose of this protocol paper is to present the design and conceptualization of a cooking intervention feasibility study among African-American adults living within a food desert to evaluate home cooking behaviors pre and post a community cooking intervention.

Based on the limited body of evidence regarding the implementation and biobehavioral outcomes of cooking interventions, the aims for the D.C. COOKS with Heart study are to:

1. Determine feasibility of delivery of cooking behavior intervention for African-American adults at risk for CVD as described by Bowen et al. (25):
  - Demand
  - Adherence to the sessions
  - Acceptability (e.g., content and delivery)
  - Practicality (e.g., mode of delivery)
  - Integration/implementation (facilitators and barriers) to home cooking
2. Determine outcomes of secondary areas of interest related to dietary quality, CVD risk factors, health behaviors, and psychosocial factors in order to gain insight into the relationship of these factors and home cooking behavior.

Table 1 shows specific primary and secondary aims for the study.

**Table 1:** Specific study aims

Phase 1	
Primary aims	a) Assess acceptability of the cooking intervention delivery and content (recipes) b) Identify facilitators and barriers to cooking frequency among focus group members
Phase 2	
Primary aims	a) Determine feasibility of the intervention, especially in association with facilitators, or barriers to cooking
Secondary aims	a) Explore the relationship between feasibility measures with intrapersonal, social and built environment factors
	b) Explore the relationship between feasibility measures with dietary quality (24 hour diet recall, Mediterranean Diet Score, Healthy Eating Index scores)
	c) Explore the relationship between feasibility measures with CVD biomarkers and anthropometric measurements

## Study design

The study design uses a feasibility study model with a mixed-method research approach. The study will occur in two phases. Phase 1 will consist of focus groups with the primary aims to identify and assess current facilitators and barriers to cooking behavior in the community to inform the study. Phase 2 will consist of the cooking intervention and clinical assessment visits at baseline and at two follow up periods, 6 weeks and 12 weeks post intervention. Overall, phase 2 will have four different time points: baseline clinical visit (TP_0_), cooking intervention (TP_1_), second clinical visit occurring 6 weeks after the intervention concludes (TP_2_), and a subsequent clinical visit occurring 12 weeks post intervention (TP_3_). The protocol for D.C. COOKS follows the SPIRIT checklist [27]. Table 2 shows the overall study plan and design.

**Table 2:** Study plan and timeline

	Phase 1		Phase 2											
Schedule of events and study plan	Enrollment for phase 1	Focus groups (community site)	Enrollment for phase 2	Clinical visit #1 TP_0_	Community cooking class—2-h session weekly for 6 weeks (community site) TP_1_						Home cooking experience	Clinical visit #2 TP_2_	Home cooking experience	Clinical visit #3 TP_3_
Week				− 4 to 0	1	2	3	4	5	6	7-11	12	13-17	18
Eligibility screen	X		X											
Informed consent		X		X										
Focus group discussion		X												
History and physical, vital signs, anthropometrics Blood draw for CVD biomarkers				X								X		X
Interview with study team				X			X			X		X		X
Dietician assessment				X								X		X
AMPS cooking skill assessment				X								X		
Daily cooking journal					X	X	X	X	X	X	X	X	X	X
Participant surveys		X		X								X		X
Community cooking intervention					X	X	X	X	X	X				

## Study population and settings

### Development of D.C. COOKS with Heart

The relevance of a study can be enhanced, and the retention of study participants improved, when community members’ knowledge and experience informs the design of the intervention and dissemination of findings [28]. Community-based participatory research (CBPR) focuses on a research topic of importance to the community and ensures that intervention strategies designed using this approach are compatible with the culture and life circumstances of the target community and population being studied [29, 30].

A previous CBPR cardiovascular health and needs assessment of a sample population from predominantly African-American churches in wards 5, 7, and 8 was conducted through the Heart Health Study in Washington D.C. to develop a community-based behavioral weight loss intervention (Protocol: 13-H-0183; NCT 01927783 PI: Powell-Wiley). The health and needs assessment screening involved measuring cardiovascular health factors within the church-based population; and evaluated social determinants of health factors that impact weight loss. The original protocol established a community advisory board, Washington, D.C. Cardiovascular Health and Obesity Collaborative Cooking Survey (DC CHOC), to consult on the planning and implementation of the assessment, as well as the interpretation and dissemination of findings from that study.

Results from the community-based cardiovascular health and needs assessment were used to inform the design and implementation of this current community-based protocol. The current study proposal and concept were presented to DC CHOC to evaluate whether members perceived the aims of the study as relevant to the community’s interest with regard to limited dietary choices and to gauge the community’s interest in intervention studies which might improve dietary behaviors among African-Americas living in wards 7 and 8. The committee provided input on intervention location and delivery, including selection of the interventionist.

Both ward 7 and ward 8 represent areas that are historically African-American, and are geographically separated from the rest of the city by the Anacostia River. While the population is diverse in economic and education levels, both communities represent significant economic and health disparities compared with the rest of the city. Both communities have the highest obesity prevalence and cardiovascular-related health events for Washington, D.C. [31], and both are considered urban food deserts. The term food desert refers to geographic areas where most people have limited access to healthy food as defined by (1) located more than half a mile from a grocery store or supermarket or (2) low rates of car access [32]. Limited access to food is a problem that affects millions of Americans every year, and these areas tend to have concentrations of low-income and minority residents, invoking socioeconomic and racial disparities. In 2016, there were 49 grocery stores in D.C. and the average number per ward was six. However, between wards 7 and 8, there are only three grocery stores for more than 140,000 residents [33]. The communities’ location within a food desert therefore limits access to fresh foods that are most often readily used for cooking, which is a limitation that may relate to participation in cooking behavior, and food purchasing choices.

### Study population

Based on the prior CBPR study, the proposed target communities for this study show a need for dietary CV risk reduction based on the risk for obesity, but also on dietary intake. In the aforementioned CBPR study, a low daily fruit and vegetable intake level of 3.2 servings per day was found compared to recommended guidelines of five per day [34]. Given the risk for CV disease, and need for improved dietary intake of fruits and vegetables, identifying ways to lower cardiovascular risk through cooking behavior within these communities may be important. Adults are the focus for this intervention and not family or child-based, because in most families adults are the nutritional decision makers [35].

Discussing health disparities requires attention and justification to how race and ethnicity are conceptualized, described, and utilized within scientific studies [36]. It is important to identify dietary quality among African-Americans, because African-Americans disproportionately live in underserved areas that are subject to social, political, and policy-related pressures that determine their food environments. Thus, the selection of African-Americans in this study stems not because unhealthy diets are intrinsic to African-Americans [37, 38], but because both wards 7 and 8 are predominately African-American, 92.4% and 92.1%, respectively [39]. Therefore, most adults who are at risk for CV disease within these communities are African-American. Moreover, within our community-based study, selection of one racial/ethnic group allows for focused insight into diet related social determinants and CVD health risks that are related to the experiences of underserved African-Americans in similar communities.

### Inclusion and exclusion criteria

Participants will be eligible for this study if they are at least 18 years of age, self-identify as African-American, English-speaking, live within the designated neighborhoods of Washington, D.C. (i.e., ward 7 or 8), and self-report a risk factor for cardiovascular disease: overweight or obese, type 2 diabetes, pre-diabetes, current or recently (12 months prior) former smoker, and hyperlipidemia.

## Methods

### Study settings

Both the phase 1 focus group and phase 2 intervention sessions will be conducted within ward 7 at a location central to both Washington, D.C. communities of interest for the study, wards 7 and 8.

The clinical setting for the study will be the National Institutes of Health (NIH) Clinical Center (CC), the U.S.’s only hospital devoted solely to clinical research. The hospital is located in Bethesda, MD, a suburb of Washington, D.C. Research participants at the NIH CC are evaluated in both outpatient and inpatient settings. For this study, an outpatient clinic utilized in the prior CBPR study will be utilized for all clinical visit appointments.

### Study procedures

In phase 1 of this study, participants (*n* = 20) will take part in one of two moderated focus groups designed to explore participants’ experiences with food and dietary selections and cooking behavior. Within this phase, participants will also take self-administered surveys related to cooking behavior, non-dietary health behaviors, and psychosocial factors. Open-ended (qualitative) questions for the focus groups were selected to elicit feedback regarding facilitators and barriers to cooking and perceptions about the feasibility of the intervention regarding (1) obtaining food for cooking, (2) use of cooking skills, (3) social norms around home cooking, (4) personal attitudes and knowledge, (5) preferences for recipes/food choice, and (6) preferences for intervention timing. Focus group feedback will also be used to modify the survey, if needed. Data analysis is anticipated to occur over a 1-month time period and the results of data analysis will inform the second phase. Specifically, the data captured from this phase will inform phase 2 in terms of recipe selection for intervention, local locations to source food for the intervention, anticipated concerns about intervention location, and schedule of sessions.

Phase 2 of the study consists of two parts: clinical visits, surveys, and interviews at pre (TP_0_) and post intervention intervals (TP_2_, TP_3_) and the cooking intervention (TP_1_). We will recruit thirty (*n* = 30) participants for this phase. Phase 2 will start with a baseline visit (TP_0_) prior to the intervention start. During this visit, a clinical history and physical examination, objective cooking assessment, dietician interview, and phlebotomy for CVD biomarkers will take place. Following completion of baseline visits for all participants, the community intervention will start with all participants who have completed the initial clinical visit. We estimate that it will take 4 weeks for all participants to complete their baseline visit. Participants will return for the second clinical visit at 6 weeks and 12 weeks post the intervention. To ensure that time of participation and days of follow up in the study are equal among participants, those who enroll and come in for their baseline visit at the earliest time point (week 4) will return for follow up visits prior to participants who completed baseline visits closer to the end of the initial time point (T_0_).

### Cooking intervention

The 6-week cooking intervention (TP_1_) in phase 2 will be delivered by a chef who is professionally trained, licensed, and certified regarding local food safety regulations. The intervention site will be a community-based kitchen site located within ward 7 or 8. Participants will be divided into two groups of 15. Each group will meet weekly at the kitchen site for a 90-min culinary education session. There will be no group assignment; participants may choose which group to attend, but can only attend one group session per week. The minimum number of participants required for delivery of the intervention will be one participant per session. In case of the need for an emergency medication administration for unknown food allergies, all sessions will be staffed with clinical research team members. Medical students trained in culinary medicine will also be present during each session to assist participants as needed. Table 3 provides an overview of the DC COOKS with Heart cooking intervention sessions.

**Table 3:** D.C. COOKS with Heart cooking intervention

Goal	Deliver cooking behavior intervention in a community setting
Type	In-person, chef-led instruction
Duration	6-week intervention with weekly 2-h sessions
Structure of sessions	Introduction and discussion of recipes and ingredients Cooking of recipes in groups Shared meal experience
Assessments	Semi-structured interviews (phone administered) Treatment fidelity Behavior change taxonomy (BCT)

### Intervention curriculum

Dietary outcomes from community cooking interventions have focused on adherence to those diet patterns related to cardiovascular health, Mediterranean diet or DASH diet. The Mediterranean diet is a dietary pattern known to reduce CV disease and mortality risk [40], presumably due to promotion of higher monounsaturated fat, fruit, and vegetable consumption. Moreover, cooking is reported as an important component of sustaining the Mediterranean diet [41]. The "Health meets Food" curriculum from the Goldring Center for Culinary Medicine (GCCM) will be used for the intervention. This longitudinal six-module curriculum is focused on the use of cooking skills to prepare foods from the Mediterranean diet. GCCM educates patients from the community in nutrition information for immediate and long-term social capital development by hands-on cooking and nutrition education [42]. The curriculum was created from prior evidence-based curricula surrounding preventive nutrition. At each session, learning objectives stem from the standard GCCM curriculum on the Mediterranean diet and nutrition education needed to start or maintain the diet. Food groups and principles of the Mediterranean diet, which include consumption of fruits and vegetables, nuts, seafood, and poultry, are highlighted in each session. The curriculum translates the Mediterranean diet for culture-specific kitchens across different socioeconomic levels [42]. In this manner, the participants are introduced to relatable food items that fall within food group categories of the Mediterranean diet. Table 4 shows a list of cooking sessions with topics, potential recipes, and study evaluation measures.

**Table 4:** Cooking intervention sessions with topics, potential recipes, and study evaluation measures per session for D.C. COOKS

Session/Lesson	Topic	Potential recipes	Study evaluation measures collected
1	Mediterranean diet: Introduction to cooking and reading recipes	Salad with red wine vinaigrette and whole grain spaghetti with meat and lentils	Cooking diaries Grocery receipts
**2**	Macronutrients: dairy, breakfast, and understanding nutrition labels	Spinach and Cheese frittata Oat pancakes	Cooking diaries Grocery receipts
**3**	Vegetables: portion sizes and lunch	One pot bean chili and tomato and cucumber salad	Cooking diaries Grocery receipts Semi-structured interview
**4**	Legumes: good shopping habits and delectable dinners	Black bean burgers with balsamic marinated mushrooms	Cooking diaries Grocery receipts
**5**	Carbohydrates and snacks	Coconut pecan date rolls and fudgy black bean brownies	Cooking diaries Grocery receipts
**6**	Fats and cholesterol	Honey mustard pork tenderloin, savory braised collard greens and strawberry salad with honey lime vinaigrette	Cooking diaries Grocery receipts Semi-structured interview

Mediterranean diet-adapted recipes in the culinary education “Health meets Food” curriculum have been utilized within a racial/ethnic and economically diverse community in the Southern U.S. In a randomized controlled trial utilizing the curriculum, a primarily African-American (75%) type 2 diabetic population showed a reduction in hemoglobin A1c up to 6 months post the intervention [43]; with 46% of the study population living in a food desert. The GCCM curriculum is also reflective of the principles of food agency [17, 44], in that participants prepare their ingredients to *mis en place* standard, work cooperatively in pairs, conversation and comparison is encouraged among pairs, and a meal is shared to engage in sensory evaluation and future meal planning. During the first session, participants will receive kitchen safety orientation provided by the chef following standard curriculum kitchen safety guidelines.

In the current protocol, recipe selection will occur in conjunction with the study team and chef interventionist along with suggestions from community members who participate in phase 1. Barriers around food sourcing that may be a result of the neighborhood food environment or location will be discussed, and when possible food sourcing for recipes will come from locations within the community.

Each intervention will consist of a discussion of a nutrition topic and of related recipes, as well as updates from prior classes including any meals/recipes made by the participants’ at home. Participants will then work in groups of three to four to prepare assigned or selected recipes. During the process of cooking the recipes, the interventionist (chef) and culinary medicine, trained medical students will assist participants with instructions or techniques, if needed. Following the cooking process, a shared meal will occur in which each group will discuss what they cooked and the interventionist/students will be available for any questions. Final clean-up of the kitchen space will occur after the shared meal.

### Intervention assessments

During the six intervention sessions, individualized semi-structured interviews will occur with participants at the mid-point (3 weeks) and end-point of the intervention (6 weeks). Responses to interviews will be used to help assess feasibility, and mechanisms of cooking behavior. Interviews maybe administered by phone to participants. At the conclusion of the intervention, the semi-structured interview will also include exit interview questions regarding the intervention only. Research staff will take field notes at all intervention sessions to evaluate treatment fidelity, delivery of information, and culinary education. In addition, intervention characteristics such as hands-on activity time versus lecture/listening time will be recorded for each session.

**Fig. 2:**
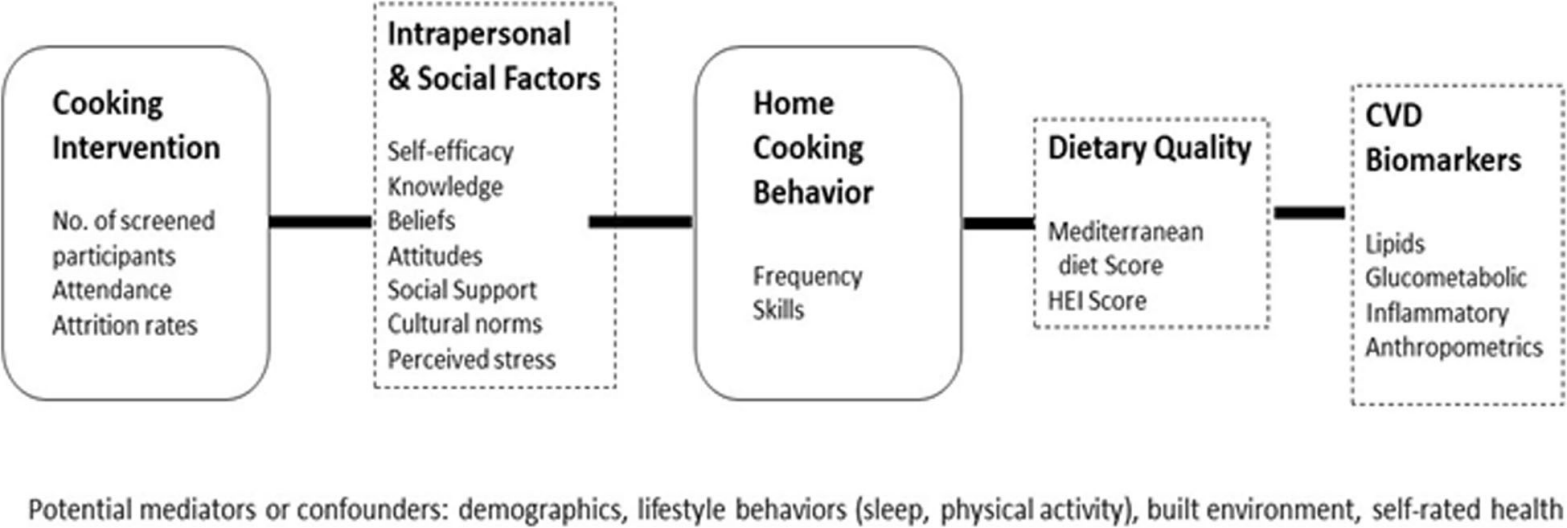
Conceptual model of potential relationships between primary and secondary outcomes

Following the principles set forth by the NIH Science of Behavior Change Program [45], interventions to change health behaviors ought to be guided by a mechanisms-of-change hypothesis. A recent review of cooking interventions by Hollywood et al. [21] found several key behavior change techniques that may contribute to dietary changes from cooking interventions: (1) information on how to perform the behavior; (2) prompt practice (prompting a person to carry out a practical task related to cooking skills or cooking behavior once or numerous times); and (3) information on the consequences of the behavior tailored to the individual [21]. As noted by identified gaps in the literature, none of the reviewed interventions measured cooking behavior as an outcome. Therefore, behavioral mechanisms for cooking behavior within cooking interventions still need to be identified. Within our study, research staff will evaluate the behavioral change techniques present in the intervention design and those presented by participants within semi-structured interviews during the cooking intervention and during post-intervention clinic follow-up visits. The rubric for the behavioral change techniques will follow those of the Coventry, Aberdeen, and London-Refined (CALO-RE) taxonomy [46] as revised by Hollywood et al [21] (Fig. 2).

## Study outcome measures

### Primary outcomes

The primary outcomes for this study are feasibility measures, home cooking behavior, and objective evaluation of cooking motor and process skills. In order to determine feasibility of the cooking intervention, we are collecting information on number of screened participants, number of participants enrolled, attendance at intervention sessions, and attrition rates. Attendance will be taken at each session. At a minimum, participants are counted as an attendee when one full intervention session has been completed. Completers of the intervention will be defined as participants who complete all six intervention sessions. Dropouts for the study will be defined as those who attend the first clinical visit, but do not attend any intervention session by the date of the final session. Attrition rates will be determined from the number of participants who stopped participating divided by the average number of participants at each time point, and over the total study period (see Fig. 4 for time points). We anticipate that our attrition rates will be at or below 33%, which is the reported attrition rate for this target population based on members of the research team’s experience recruiting from wards 7 and 8 [34]. Feasibility success will be achieved if the majority of participants (> 50%) reach 50% participation (3/6 sessions). This is based on two reports in the literature of cooking intervention studies among African-Americans in which this attendance metric was met [13, 15]. Information collected on feasibility and cooking behavior and skills will allow for appropriate sample size calculations for future studies.

Cooking behavior will be determined through multiple variables to provide a convergence of data. Using paper cooking diaries, participants will be instructed to start daily recording of home cooking following the initial clinical visit and will stop after the last visit, for a total collection time of 18 weeks. The design for the cooking diaries is fixed-schedule (once per day). Each daily entry will consist of marking the frequency of cooking at home, as well as commenting on enjoyment, interest [47], and perception of cooking skills.

**Fig. 3:**
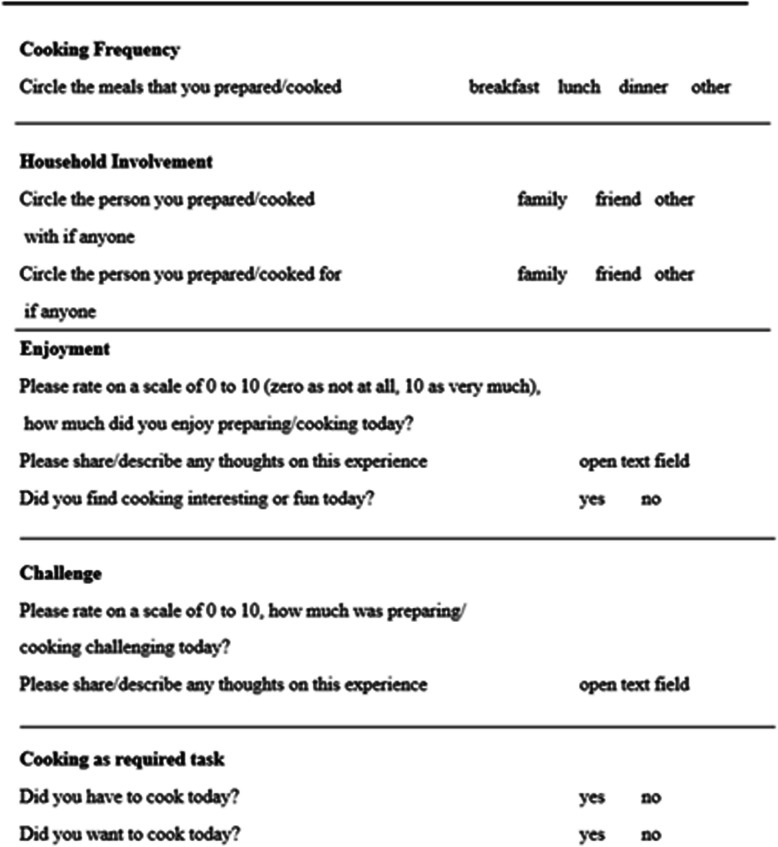
Cooking diary components by topics. Participants will be instructed to respond for each day of the week

Figure 3 illustrates the questions and answer choices for each cooking diary entry. At each clinical visit (TP0, TP2, TP3), additional measures of cooking behavior will be collected. Home cooking frequency, family meal behavior, and frequency of purchasing foods outside of the home over the prior 7 days will be determined using standardized national survey questions [48, 49]. Self-efficacy for cooking and food agency will be determined from validated survey tools [14, 44]. Psychosocial determinants of cooking will be assessed with a survey tool pre-tested within the target community by our team [50].

As stated earlier, a current gap in the literature is whether participation and engagement level in cooking behavior is expected to vary by participant’s baseline cooking skill. To our knowledge, assessment of baseline cooking skill has not been described in published cooking intervention studies as a participant characteristic. Assessment of Motor and Process Skills (AMPS) is a kitchen performance assessment test delivered by occupational therapists to assess an individual’s performance skills (motor and process). For each task, motor and process skills are evaluated. Motor skills involve hand movement and coordination. Process skills involve the actions used to organize and adapt behavior over time in order to complete a task. The AMPS test will be administered by trained occupational therapists in the Department of Rehabilitation kitchen at the NIH Clinical Center at baseline and the 6-week clinical visit. Analysis of AMPS will be done with a computerized scoring system where the correction for the score is based on skilled item difficulty, task challenge, and rater severity [51]. Because the test encourages participants to choose familiar tasks, it is participant centered. Additionally, AMPS has been validated for use in an African-American adult population [52].

### Secondary outcomes

Health behaviors are often socially patterned [67]. Moreover, dietary behaviors are likely influenced by tradition and a shared history that is passed from generations which then determine intrapersonal (taste preferences), interpersonal (cultural and familial paradigms), and community set norms [67]. Therefore, secondary outcomes measured are related to psychosocial factors, the neighborhood food environment, dietary intake, and health behaviors, such as physical activity and sleep quality. Figure 2 illustrates our conceptual model of potential relationships between primary and secondary outcomes. Table 5 provides a list of primary and secondary study measures with instrument type and description of measurement.

**Table 5:** Table of study measures with instrument type and description of measurement

Measurement	Type of instrument(s)	Description
Primary outcomes		
Facilitators and barriers to cooking		
Cooking diaries	Daily self-administered data collection	Daily responses to cooking frequency questions will be used
Cooking self-efficacy scale (CSES) [14, 53]	Self-administered 7 item instrument	CSES assesses the degree of confidence in performing basic cooking activities on a 5-point Likert scale
Psychosocial factors related to cooking [50]	Self-administered 32 questions (61 items) D.C. CHOC Cooking Survey	D. C. CHOC is a self-administered 32 question (61 items) survey to assess psychosocial determinants and developmental exposure to cooking as well as confidence for certain cooking techniques and food shopping. It will also capture cooking frequency over the last 7 days.
Food agency [17]	Self-administered 28 item Cooking and Food Provisioning Action Scale (CAFPAS)	CAFPAS is a 28 item instrument with 3 sub-scales ( food self-efficacy—13 items, food attitude—10 items, perceived influence of non-food barriers on provisioning—5 items). The scale has undergone face and construct validity and reliability testing, with Cronbach’s alpha = 0.7
Cooking skills [51]	Assessment of Motor and Process Skills (AMPS)	AMPS is a kitchen performance assessment test delivered by occupational therapists to assess an individual’s performance skills. The AMPS will be conducted at baseline and 6-week clinic visit.
Feasibility measures		
Attrition	Number of participants that complete the study	Attrition will be based on the number of participants at the start and remaining at the end of the study, as well as at each time point. Attrition rates will be determined from the number of participants who stopped participating divided by the average number of participants at each time point, and over the total study period
Attendance	Attendance record at each intervention session using study log	Attendance will be taken at each intervention session and rates will be analyzed to determine the desired dosage (how much, how often and at what interval) for each participant by their characteristics
Participant burden	Observations of research team and participant feedback	Participant burden will be determined by data collection assessments, research team’s perception of participants’ understanding of questions and data collection methods, and if participants respond with missing or unusable data. The study team will also assess if participants have enough time and capacity to complete data collection procedures.
Treatment fidelity [54]	Guidelines for treatment fidelity from the NIH Behavior Change Consortium workgroup on treatment of fidelity	Treatment fidelity assessment grid will be used to determine implementation of the intervention. Cooking diaries and interviews will also be used as a measure of implementation/intervention fidelity.
Secondary outcomes		
Social network index [55, 56]	Self-administered 12 item measure	SNI is a self-administered 12-item instrument that assesses participation in 12 types of social relationships. There are three measures within the SNI: number of high-contact roles (network diversity), number of people in social network, and number of embedded networks.
Health promoting Lifestyle Profile II (HPLP-II) [57]	Self-administered 52-item instrument	The HPLP-II is a self-administered 52-item instrument that measures the frequency of self-reported healthy behaviors. It consists of 6 subscales: physical activity, spiritual growth, health responsibility, interpersonal relations, nutrition, and stress management (including sleep quality).
Perceived stress [58]	Perceived stress scale (PSS) is a self-administered 10 item instrument	PSS measures an individual’s perceptions about the nature of events and their relationship to coping resources of that individual. This 10 item tool uses a 5 point Likert scale for each item.
Neighborhood factors		
MESA Neighborhood Perception of Healthy Food Availability Scale [59]	Self-administered shortened 3 item scale (the original being 6-item).	This scale is used to calculate perceived healthy food availability in the neighborhood, which is defined as within a 20-min walk or one mile distance from the individual’s home.
Perception of Neighborhood Food Retail Outlets [60]	Self-administered 9 item questionnaire	This tool consists of 9 items and tests types of retail outlets available within the neighborhood, which is defined as within a 20-min walk or one mile distance from the individual’s home.
Neighborhood satisfaction [61]	Single question with 5 answer choices	Neighborhood satisfaction will be measured with the question, “All things considered, would you say you are very satisfied, satisfied, dissatisfied, very dissatisfied, or neutral - neither satisfied nor dissatisfied with your neighborhood as a place to live?”
Food purchasing practices [61]	Grocery receipts	Grocery receipts will be collected at intervention sessions and follow up CC visits, to assess overall dietary quality and utilization of food store type
	Food purchasing practices	Food purchasing questionnaire measures frequency of major food shopping with 11 different types of store options. It also inquires about mode of transportation for that major food shopping trip.
	Food Away from Home frequency	Food away from home will be assessed by one question from CD-NHANES-DBQ, 2015 (During the past 7 days, how many meals did you get that were prepared away from home in places such as restaurants, fast food places, food stands, grocery stores, or from vending machines?), whereas the meal could mean breakfast, lunch or dinner.
Self-rated health [62]	Self-administered 1 item measure	Self-rated health is assessed through one question and it measures the general health state or change in state which could be associated with outcomes of interest
Sleep quality assessment [63]	Pittsburgh Sleep Quality Index (PSQI)	PSQI is a 9-item self-administered measure that assesses the quality and patterns of sleep. PSQI has seven subscales and altogether they create a total score of sleep quality.
Physical activity [64]	International Physical Activity Questionnaire (IPAQ)-Short form	IPAQ short form is a self-administered 7 item measure that assesses the types and intensity of physical activity and also the time spent while sitting.
HEI & Mediterranean diet adherence screener [65, 66]	24-h food frequency questionnaire	A nutritional assessment will be done by a member of the dietician team from the CC Nutrition Department using 24 hour dietary recall. This will provide information regarding dietary patterns and eating behaviors. Study staff (registered dieticians) will analyze the food records using Nutrition Data System for Research (NDS-R) software for energy, protein, carbohydrate, fat, alcohol, caffeine, and micronutrient intake. A composite diet quality score (a measure of nutritional status and adherence to dietary guidelines) will be calculated using the Healthy Eating Index.
	14-item Mediterranean diet adherence screener (MEDAS)	MEDAS is a 14 item questionnaire that assesses adherence to the Mediterranean diet. Photographs of portions and serving sizes are used to facilitate accurate completion. Validation of the MEDAS questionnaire and test-retest reliability for English version has been conducted.
Family meals ()	Family Meal frequency	Family meal socialization will be assessed by one question from CDC-NHANES-DBQ 2015 (During the past 7 days, how many meals did all or most of your family sit down and eat together at home?), where the meal could mean breakfast, lunch or dinner
CVD biomarkers	- BMI - Blood pressure - A1C - Lipid screen - CBC with differential - Glucose (fasting) - Insulin (fasting) - Advanced lipid panel (fasting) - CRP, IL-6	CVD biomarker collection duet to known role of dietary behaviors on CVD risk factors
Anthropometrics	Waist circumference and waist to hip measurement	Waist circumference (at the top of the iliac crest) and hip circumference (at the maximum protuberance of the buttocks) will be measured in triplicate with the average of measurements used as data for clinical visit time point

All secondary outcomes will be obtained through self-administered questionnaires, except for dietary intake. Dietary intake will be measured using a 24-h recall of food and drink intake collected by the nutrition team from the NIH Clinical Center Nutrition Department. During the interview, the nutrition staff member will ascertain the content and quantity of dietary intake, as well as inclusion of home cooked versus purchased foods. This will be done to determine baseline and post intervention diet quality of home cooked food, not just general diet quality. Data from the 24-h recall will also provide information regarding dietary patterns. Study staff (registered dieticians) will analyze the food records using Nutrition Data System for Research (NDS-R) software for energy, protein, carbohydrate, fat, alcohol, caffeine, and micronutrient intake [68]. A composite diet quality score ranging from 0 to 100 will be calculated using the Healthy Eating Index, HEI-2015 (https://epi.grants.cancer.gov/hei/) [65]. Calculated HEI score assesses the extent to which a participant follows the 2015–2020 Dietary Guidelines for Americans (DGA). Because the HEI-2015 aggregates dietary components, it provides a hypothesis-oriented approach to determine participants’ dietary pattern. The Mediterranean Diet score will be determined by the Mediterranean Diet Adherence Screener (MEDAS) [66].

### Recruitment and retention plan

For phase 1, focus groups will be completed prior to the beginning of phase 2. Recruitment will utilize participants from the community-based participatory research study (NCT01927783) for the focus groups, as well as adults associated with collaboration sites of the DC CHOC. Initial contact will occur through phone contact with identified eligible participants. Likewise, email announcements will be sent to collaboration sites. Recruitment of focus group participants will use a purposive snowballing sample of African-American adults (age > 18) who live in wards 7 or 8 in Washington D.C. However, we will aim for the study recruitment to lead to variation in demographic subgroups. Identified participants may or may not be currently enrolled in or participated previously in a community cooking education program/class. At the time of phone contact with potential participants, study staff will ask screening eligibility questions regarding self-reported risk for cardiovascular disease. If potential participants are deemed eligible based on aforementioned inclusion/exclusion criteria, they will be invited to participate in a focus group.

**Fig. 4:**
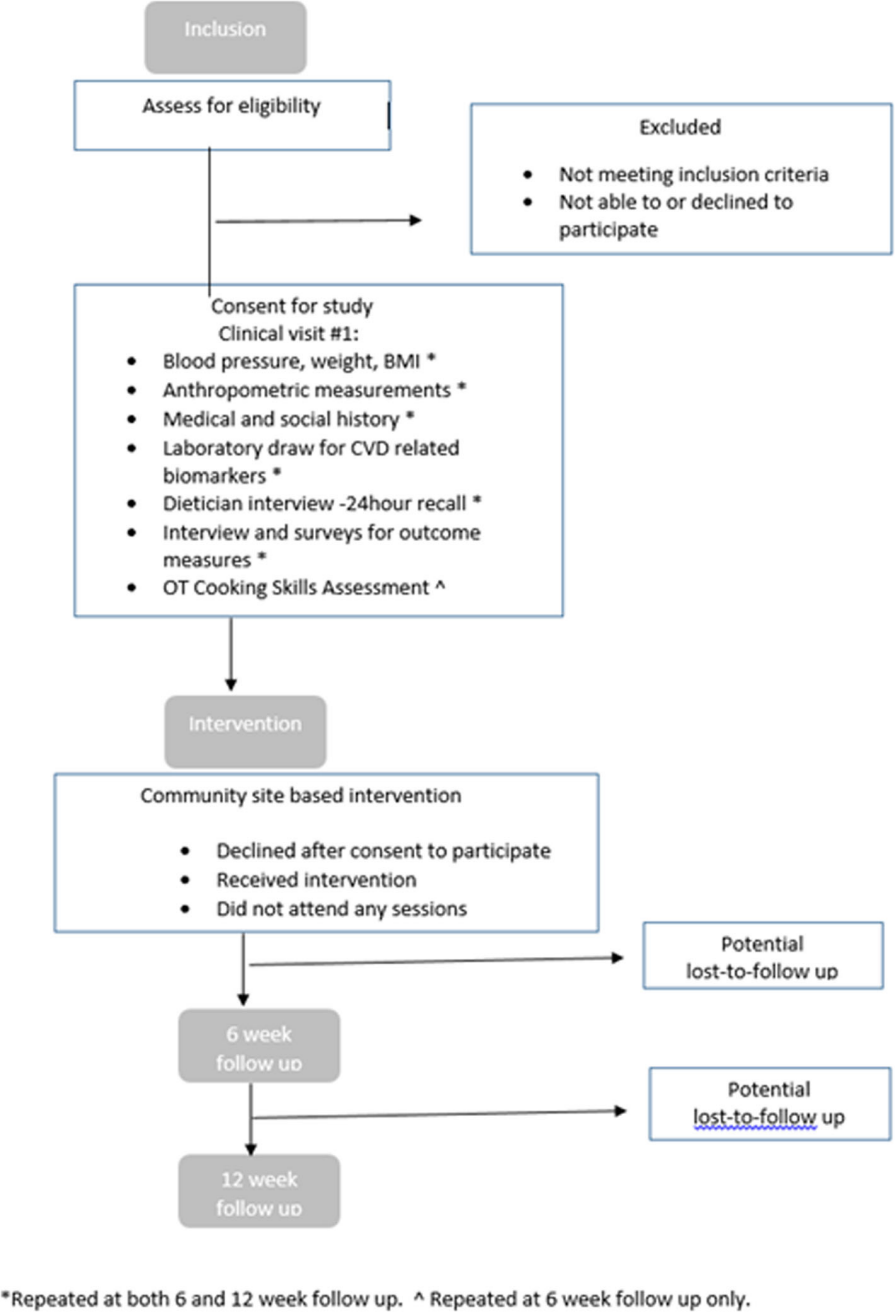
The planned flow and steps of data collection within phase 2 relevant to expected recruitment and retention

For phase 2, similar strategies for identifying participants will be used as in phase 1. At the time of contact with potential participants, study staff will ask screening eligibility questions regarding self-reported risk for cardiovascular disease. If potential participants are deemed eligible based on aforementioned inclusion/exclusion criteria, they will be invited to schedule a baseline visit. Signing of informed consent document for the protocol will be obtained at clinical visit #1 (TP_0_) after discussion and explanation of study requirements with a member of the research study team. Eligible study participants will have consent document mailed to them to review prior to clinical visit #1*.* The planned flow and steps of data collection within phase 2 relevant to expected recruitment and retention are illustrated in Fig. 4.

Retention efforts focused on participant engagement include reminder phone calls to participants on a weekly basis during the intervention and 1 week and 1 day prior to all clinical visit appointments. Participants will be discontinued from the study if any of the following should occur: participant requests to be removed from the study, participant does not participate in the initial clinical visit, or participant is unable to participate in the intervention component.

## Analysis

### Qualitative data

A phenomenological methodological approach will be undertaken in both phase 1 and 2 to understand participants’ lived experiences with cooking at home within the context of their neighborhood and household. Phase 1 will focus on gathering an understanding of non-intervention based conditions. While in phase 2, we will gather an understanding of the participation experience within the specific setting of the cooking intervention. In both phases, qualitative data collection and analysis will use the four criteria that comprise the rigorous standards for qualitative research: credibility, dependability, confirmability, and transferability [69]. Credibility will be maintained by the independent analyses of our transcribed interview data to ensure that our theme categories cover all relevant data. Credibility will be further strengthened through the consensus-building process, which will ensure that similarities within and differences in our theme categories are thoroughly evaluated. Dependability will be established by maintaining facilitator consistency through data collection and through data analysis by our team. Confirmability will be supported by developing an electronic, thematic database and through the preservation of interviewer perceptions via interview/focus group field notes. Our diverse study team, comprising individuals with various clinical and research-related expertise with members of the community, will support the criteria of transferability.

In phase 1, the sources for qualitative data are the transcripts and research team field notes from the focus groups. In phase 2, the source of the qualitative data will come from semi-structured interviews conducted at all study time points. Analysis in both phases will use the de-identified verbatim transcription of audiotapes and field notes written by research team members. Members of the research team will develop a codebook based on themes from the interviews. Each thematic code will be accompanied by an operational definition that will allow for clarity and consistency in the coding process. A team of coders will independently review all transcripts. Discordant coding will be discussed until consensus among the coding team is achieved. Once the iterative process of consensus building is complete, an intramural expert in qualitative methodology will validate the final themes and coding. After data are coded, NVivo will be utilized for data management.

### Quantitative data

#### Sample size justification

For phase 2, sample size justification was done in accordance with Billingham et al. [70]. From the literature for published cooking interventions, intervention group size ranges from 12 to 30 participants [13–15, 43]. We therefore selected a sample size of 30 participants for phase 2. We also based the sample size on expected attrition. From our previous community study (NCT 0192778 [34];), we anticipate a 33% attrition rate for the first clinical visit. Therefore, in order to reach 30 participants, we plan to screen 45 participants*.*

#### Data analysis

Baseline demographic and clinical data will be analyzed using descriptive statistics. Categorical data will be presented as frequencies and percentages, while continuous data will be presented as means and standard deviations. Patterns of missing data will be examined thoroughly to assess whether any questions were systematically skipped by all participants or any sub-group of participants.

All continuous variables of interest for quantitative data analysis will have baseline evaluation done to check for outliers and normal distribution. Data determined to not have normal distribution will be expressed as medians with interquartile ranges. If the assumptions of normality are not violated, we will proceed with parametric testing. If the assumptions of normality are violated, non-parametric tests will be utilized. For quantitative analyses, statistical significance will be considered at two-tailed, *p* < 0.05. Statistical analysis will be conducted in available statistical software (for example SAS, SPSS). Inferential statistics will be conducted for the purposes of exploration, but without placement of conclusions on the effectiveness of the intervention. Relationships between variables will be examined with bivariate correlation coefficients (for two continuous variables), Chi-squares (for two categorical variables), and *t* tests. Analysis of variance (ANOVA) (for one categorical and one continuous variable) will be conducted to explore sub-group analysis. Linear or logistic regression analyses, with multivariate analysis when applicable, will also be pursued to examine determinant models of cooking behaviors or feasibility measures.

Phase 2 consists of four time points. Group level analyses will be conducted with reporting and comparison of group means at each time point. Within-person analysis using repeated measures will also be done to compare within person changes across time points, including repeated measures ANOVA and *t* tests where applicable.

For the survey responses related to the primary outcomes, facilitators, and barriers to cooking (DC CHOC, CAFPS, CES), question items deemed as positively related to facilitating cooking will have a sum of scores calculated and then divided by total number of respondents. Survey items deemed as negatively related to cooking (barriers) will be reverse scored to determine mean scores. Within DC CHOC Survey, responses for cooking perception will be dichotomized as agreement with cooking if response is ≥ 5, as reported in Wolfson 2016 [71].

From the cooking diaries, daily cooking frequency amounts will be aggregated into cooking frequency for each week of the study, the average at each time point of phase 2 for each participant. Cooking frequency at each time point will also be dichotomized based on the average cooking frequency per week of 10.8 out of 21 possible meals, as reported in a national survey of U.S. adults [71].

## Data collection and management

All analyzed data will have unique, study-specific numerical identifiers so that research data can be attributed to an individual human subject participant. Data obtained during the conduct of the protocol will be kept in secure password-protected network drives or in approved alternative sites that comply with NIH security standards. This includes recorded participant interviews. The principal investigator and associate investigators, research nurses, and/or contracted data managers will assist with the data management efforts.

Survey measures will be collected electronically on clinic tablets used by participants on clinical visit days. Laboratory data and clinical examination data will be collected using the Clinical Center electronic health record known as Clinical Research Information System (CRIS). Cooking diary data will be completed by paper format and then entered electronically and managed using REDCap electronic data capture tools hosted within the Biomedical Translational Research Information System (BTRIS) at the NIH. REDCap (Research Electronic Data Capture) is a secure, web-based application designed to support data capture for research studies [72]. REDCap was developed and is licensed by Vanderbilt University. All use of REDCap will occur in compliance with NIH security standard. Participants without a personal electronic device will have paper cooking diaries provided for mail return to the research team in pre-stamped, self-addressed envelopes. The diaries will then be entered using a double-data entry method into a secure data management system.

Data from enrolled subjects will be stored until they are no longer deemed of scientific value or if a subject withdraws consent for their continued use, at which time they will be destroyed. Should we become aware that a major breach in our plan for tracking and storage of data has occurred, the Internal Review Board (IRB) will be notified. Each NIH protocol undergoes a yearly departmental, independent audit in addition to yearly continuing reviews by the IRB.

Data management committees (DMC) are not usually warranted in early studies such as pilot/feasibility studies, but formal monitoring groups may be useful for certain types of early clinical studies [73]. For this protocol, a study monitoring group consisting of study investigators will be formed and meet regularly throughout the study time frame. Safety monitoring will occur following standard procedures within the NIH. These procedures include reporting of adverse events related to participation in the study. Adverse events will be reported to the principal investigator (PI) with recommendations and follow up, as well as documentation in the clinical electronic patient record. Adverse events will focus on ones related to the intervention and clinical visits where the NIH is involved either directly or indirectly by recommending certain interventions. Adverse events associated with the cooking intervention will be monitored by the PI, serving as safety officer, and will be reported to the IRB as appropriate and as part of the annual report.

### Evaluation of risks/discomforts and benefits ratio

This protocol will assess feasibility of administering a cooking behavior intervention in a community at risk for CVD within a food desert. Participation in phase 2 may confer a health-behavior, nutrition education based benefit to participants. However, it is possible that there may be no direct benefit. It is also possible that in both phase 1 and phase 2, answering survey questions may cause discomfort to participants. Furthermore, in phase 2 phlebotomy for laboratory study collection may lead to discomfort at the puncture site. If this should occur, every effort will be made to address and minimize participant discomfort.

### Compensation

Participants will be compensated for research-related discomfort and inconveniences in accordance with NIH guidelines. If participants are unable to finish the study, they will be paid only for those parts completed.

### Dissemination of results

This protocol was developed in collaboration with the DC CHOC community advisory board. Therefore, results of the study germane to the primary and secondary outcomes as well as results determined to be related to stated dietary behavior concerns voiced by the committee, will be presented at the DC CHOC committee meetings. Publications related to baseline data and primary outcomes will be disseminated to study participants once published.

Since the protocol is federally funded, any manuscript to be published in a peer-reviewed journal will be submitted to PubMed Central or public access upon acceptance for publication.

## Study strengths and limitations

A key strength of our study is the mixed methods design, with a sequential approach in allowing the qualitative data from phase 1 inform phase 2, and a simultaneous approach in phase 2 using both quantitative and qualitative data. Mixed methods studies are noted as important for understanding health disparities [74] and health behaviors [75]. Another strength is the use of an objective cooking skill assessment and use of over lapping measures of behavior (the primary outcome). The study’s other strength is community recruitment and involvement which can help to create pragmatic results relevant to members of the community. Despite its strengths, there are some limitations to consider. Reliance on a previously identified group of participants for recruitment may limit our external validity to other members of the community who have not previously participated in community research. The use of self-reported data for secondary outcome measures that can be influenced by biases such as recall or social desirability, serves as an additional limitation.

## Conclusions

With dietary guidelines focusing on foods that require cooking and preparation, such as fruits and vegetables, it is important to identify dietary behaviors which may promote adherence to current guidelines. Furthermore, for communities disproportionately affected by diet-related chronic diseases, identifying dietary behaviors that may offer chronic disease prevention through better guideline adherence is significant. This feasibility study seeks to address a critical gap in our understanding of how cooking interventions may lead to cooking behavioral changes and what factors (intrapersonal or interpersonal) may facilitate or impede needed behaviors at home in order to follow cardiovascular preventive diets.

## Trial status

This study was approved by the NIH Intramural Institutional Review Board in February of 2020 (NCT 04305431). We expect to begin recruitment in late Summer or early Fall of 2020.

## Supplementary information

**Additional file 1.** SPIRIT 2013 Checklist: recommended items to address in a clinical trial protocol and related documents.

## Data Availability

Not applicable.

## Abbreviations

A1C: Glycated hemoglobin
AA: African-American
AMPS: Assessment of Motor and Process Skills
ANOVA: Analysis of variance
Apo: Apolipoprotein
BMI: Body mass index
BP(s): Blood pressure
CAPFS: Cooking and Food Provisioning Action Scale
CBC: Complete blood count
CBPR: Community-based participatory research
CC: Clinical Center
CALO-RE: Coventry, Aberdeen, and London-Refined
CRIS: Clinical Research Information System
CSES: Cooking Self-efficacy Scale
CVD: Cardiovascular disease
DASH: Dietary approaches to stop hypertension
DC CHOC CS: DC Cardiovascular Health and Obesity Collaborative Cooking Survey
DGA: Dietary Guidelines for Americans
DMC: Data Management Committee
FDA: Food and Drug Administration
HEI: Health Eating Index
HHS: Health and Human Services
HPLP-II: Health Promoting Lifestyle Profile-II
hs-CRP: High-resolution, C-reactive protein
IL-6: Interleukin 6
IPAQ-SF: International Physical Activity Questionnaire–Short Form
IRB: Internal Review Board
MEDAS: Mediterranean Diet Adherence Score
NDS-R: Nutrition Data System for Research
NIH: National Institutes of Health
NIHCC: National Institutes of Health Clinical Center
OT: Occupational therapist
PBC: Perceived behavioral control
PI: Principal investigator
PII: Personally
PQSI: Pittsburgh Sleep Quality Assessment
PSS: Perceived Stress Scale
REDCap: Research Electronic Data Capture
SCT: Social cognitive theory
SNI: Social Network Index
TP: Time point
TPB: Theory of planned behavior
